# Integrated analysis of spatial transcriptomics and CT phenotypes for unveiling the novel molecular characteristics of recurrent and non-recurrent high-grade serous ovarian cancer

**DOI:** 10.1186/s40364-024-00632-7

**Published:** 2024-08-12

**Authors:** Hye-Yeon Ju, Seo Yeon Youn, Jun Kang, Min Yeop Whang, Youn Jin Choi, Mi-Ryung Han

**Affiliations:** 1 Division of Life Sciences, College of Life Sciences and Bioengineering, Incheon National University, Incheon, 22012 Korea; 2 Department of Radiology, Seoul St. Mary’s Hospital, College of Medicine, The Catholic University of Korea, Seoul, 06591 Korea; 3 Department of Hospital Pathology, Seoul St. Mary’s Hospital, College of Medicine, The Catholic University of Korea, Seoul, 06591 Korea; 4 Department of Obstetrics and Gynecology, Seoul St. Mary’s Hospital, College of Medicine, The Catholic University of Korea, Seoul, 06591 Korea; 5 Cancer Research Institute, College of Medicine, The Catholic University of Korea, Seoul, 06591 Korea; 6 Institute for New Drug Development, College of Life Science and Bioengineering, Incheon National University, Incheon, 22012 Korea

**Keywords:** High-grade serous ovarian cancer, Spatial transcriptomics, Radiogenomics, Tumor microenvironment

## Abstract

**Background:**

High-grade serous ovarian cancer (HGSOC), which is known for its heterogeneity, high recurrence rate, and metastasis, is often diagnosed after being dispersed in several sites, with about 80% of patients experiencing recurrence. Despite a better understanding of its metastatic nature, the survival rates of patients with HGSOC remain poor.

**Methods:**

Our study utilized spatial transcriptomics (ST) to interpret the tumor microenvironment and computed tomography (CT) to examine spatial characteristics in eight patients with HGSOC divided into recurrent (R) and challenging-to-collect non-recurrent (NR) groups.

**Results:**

By integrating ST data with public single-cell RNA sequencing data, bulk RNA sequencing data, and CT data, we identified specific cell population enrichments and differentially expressed genes that correlate with CT phenotypes. Importantly, we elucidated that tumor necrosis factor-α signaling via NF-κB, oxidative phosphorylation, G2/M checkpoint, E2F targets, and MYC targets served as an indicator of recurrence (poor prognostic markers), and these pathways were significantly enriched in both the R group and certain CT phenotypes. In addition, we identified numerous prognostic markers indicative of nonrecurrence (good prognostic markers). Downregulated expression of *PTGDS* was linked to a higher number of seeding sites (≥ 3) in both internal HGSOC samples and public HGSOC TCIA and TCGA samples. Additionally, lower *PTGDS* expression in the tumor and stromal regions was observed in the R group than in the NR group based on our ST data. Chemotaxis-related markers (*CXCL14* and *NTN4*) and markers associated with immune modulation (*DAPL1* and *RNASE1*) were also found to be good prognostic markers in our ST and radiogenomics analyses.

**Conclusions:**

This study demonstrates the potential of radiogenomics, combining CT and ST, for identifying diagnostic and therapeutic targets for HGSOC, marking a step towards personalized medicine.

**Supplementary Information:**

The online version contains supplementary material available at 10.1186/s40364-024-00632-7.

## Introduction

High-grade serous ovarian cancer (HGSOC) is the most frequent and fatal malignancy among gynecological cancers, with a 10-year survival rate of less than 30% [1, 2]. Due to the high recurrence and metastasis rate, about 80% of patients tend to recur despite proper management (surgery and chemotherapy) [3]. HGSOC is histologically heterogeneous and is diagnosed in the advanced stage (III-IV) [3, 4]. Therefore, it has been generally assumed that there is an urgent need to better understand the HGSOC tumor microenvironment (TME), and the role of the TME in the context of recurrence and chemoresistance [5]. However, previous studies have been limited by the lack of detailed research on HGSOC TME in terms of integrating spatial transcriptomics (ST) with radiomics analysis.

The recent ST technology allows us to interpret distinct cell populations correlated with their spatial distribution and cellular interactions [6]. Differentially expressed genes (DEGs) by single cell or spatial location that could not be identified by bulk RNA sequencing (bulk RNA-seq) can be analyzed using single-cell RNA sequencing (scRNA-seq) or ST, respectively [7]. ST revolutionizes promising ways to understand the spatial context and cellular heterogeneity in TME because it enables gene expression profiling while preserving tissue location information [8].

Computed tomography (CT) is part of the standard of care used to propose surgical debulking and evaluate chemotherapy responses in patients with HGSOC [9]. Radiogenomics, an analysis of the association between imaging and genetic features, may be useful for understanding the heterogeneity and discovering biomarkers for the precise treatment of HGSOC [10–12]. Nevertheless, interpreting the heterogeneity of HGSOC through radiogenomics analysis that combines CT and ST has not yet been explored. HGSOC contains a wide range of non-malignant cell types, and a recently developed technology, scRNA-seq, has made it possible to explain the TME of HGSOC, which consists of various cell types [13]. ST technology that captures genome-wide readouts across tissue space plays an important role in figuring out how genes are spatially expressed in complex TME [7]. Integrative analysis of scRNA-seq and ST enables the differentiation of tumor, stroma, and immune regions of HGSOC and investigation of ligand–receptor (LR) interactions by tissue location, which can reveal the importance of prognostic prediction [14].

Interestingly, ovarian cancer tends to spread easily to the peritoneum [15]. According to the American Joint Committee on Cancer (AJCC) 8th, primary peritoneal carcinomatosis without primary ovarian mass uses the same staging system as ovarian cancer [16]. In prior radiogenomics research on HGOSC, the genetic examination of the primary ovarian tumor was analyzed in correlation with the features of peritoneal seeding tumors observed in CT images [9, 12, 17]. Therefore, in this study, we determine the CT phenotype including the characteristics of the primary ovarian mass and peritoneal tumor.

In this study, we analyzed the spatial genomics and CT image data of eight patients with HGSOC to identify the intratumoral and intertumoral heterogeneity of recurrent HGSOC. We simultaneously analyzed public HGSOC scRNA-seq data and public HGSOC bulk RNA-seq data to supplement and validate our results. Through a comprehensive analysis, DEGs for each CT imaging feature were identified according to the location of the tissue. The current study aimed to provide an in-depth understanding of how to improve the prognosis of recurrent HGSOC.

## Methods

### Patients

All specimens from patients with cancer were obtained with appropriate consent and approval from the institutional review board of Seoul St. Mary’s Hospital, The Catholic University of Korea, College of Medicine (KC22SISI0687). We obtained ovarian cancer tissues from eight treatment-naïve patients who underwent cytoreductive surgery. ST analysis was performed on the ovarian cancer tissues. The clinicopathological data are presented in Table 1. Subgroup analysis was performed among the eight patients with HGSOC: five in recurrent (R) and three in non-recurrent (NR) groups. Recurrence was defined as the presence of newly developed lesion(s) on CT six months after the last treatment. Individuals not experiencing recurrence within 60 months (5 years) were categorized as “non-recurrent”.

**Table 1:** Patients’ clinicopathologic characteristics

Patients' ID	Age	Pathology	Stage (FIGO)	Recurrence	BRCA1/2 status	Initial CA125 (IU/mL) (normal range < 35)	CA125 at recurrence (IU/mL) (normal range < 35)	Survival (mo)	Expired / alive	Concurrent cancer	Family history of cancer	Pathologically proven primary site	Pathologically proven metastatic sites in abdomen
R1	45	High-grade serous carcinoma	III	Yes	Negative	546	653	71	Alive	Thyroid cancer	No	Left ovary	Peritoneal carcinomatosis, pelvic and para-aortic lymph node
R2	61	High-grade serous carcinoma	III	Yes	Negative	2723	248	60	Expired	No	No	Left ovary	Peritoneal carcinomatosis, pelvic and para-aortic lymph node
R3	49	High-grade serous carcinoma	IV	Yes	*BRCA1* mut	990	305	56	Expired	Breast cancer	Sister: gastric cancer	Right ovary	Peritoneal carcinomatosis, pelvic and para-aortic lymph node
R4	49	High-grade serous carcinoma	IV	Yes	*BRCA1* mut	4220	254	40	Expired	No	Mother: ovarian cancer	Both ovaries	Peritoneal carcinomatosis, pelvic and para-aortic lymph node
R5	34	High-grade serous carcinoma	III	Yes	*BRCA1* mut	1894	63	81	Alive	No	No	Both ovaries	Peritoneal carcinomatosis, pelvic and para-aortic lymph node
NR1	51	High-grade serous carcinoma	III	No	Negative	1050		90	Alive	No	Father: lung cancer	Both ovaries	Peritoneal carcinomatosis
NR2	65	High-grade serous carcinoma	IV	No	Negative	127		78	Alive	No	Mother: gastric cancer	Left ovary	Peritoneal carcinomatosis
NR3	64	High-grade serous carcinoma	III	No	*BRCA2* mut	3756		63	Alive	No	No	Both ovaries	Peritoneal carcinomatosis, pelvic and para-aortic lymph node

*Abbreviation*: *FIGO* International Federation of Gynecology and Obstetrics, *CA125* cancer antigen 125, *mo* months

### Spatial library construction and sequencing

Visium Spatial Gene Expression Solution was used to measure total mRNA in the tissue sections and locations where gene activity occurred (10 × Genomics). The tissue sections were fixed, stained on Visium Spatial Tissue Optimization Slide, and permeabilized. cDNA was generated using fluorescently labeled nucleotides and was visualized. Ultimately, the tissue was enzymatically eliminated, resulting in fluorescently labeled cDNA, which could be observed by fluorescence microscopy to determine the optimal permeabilization duration.

Libraries were prepared according to the Visium Spatial Gene Expression Guide CG000239 (10 × Genomics, Pleasanton, CA, USA). The tissue sections placed on Visium Spatial Gene Expression Slide were fixed and stained, and cellular mRNA was captured using primers at gene expression spots. Following the transfer of cDNA from the slide, the spatially barcoded full-length cDNA was amplified by PCR to generate the required mass for library construction. Purified libraries were quantified by qPCR according to the KAPA qPCR Quantification Protocol Guide (Roche Sequencing Solutions, Pleasanton, CA, USA), and their quality was assessed using Agilent Technologies 4200 TapeStation (Agilent Technologies, Santa Clara, CA, USA). Libraries were sequenced on the NovaSeq platform (Illumina, San Diego, CA, USA).

### ST data processing

10 × Genomics Space Ranger v2.0.0 was used to perform gene counting of the Visium sequencing data. Fastq files were processed using the GRCh38 reference, and raw gene expression matrices were used for downstream analyses. The count matrix and image data were further analyzed using the R package Seurat v4.4.0 [18]. Each sample was loaded individually. The Seurat v4.4.0 SCTransform function was used to perform data normalization [19]. The regressing out of the percentage of mitochondrial and ribosomal counts was conducted. To reduce the dimensionality of the data, principal component analysis was performed using the RunPCA function, and the percentage of the variation associated with each principal component (PC) was calculated. The number of significant PCs for each sample was determined by selecting a metric with a “variation change rate of less than 0.1% between consecutive PCs and metric” or “PCs contribute only 5% of the standard deviation and the PCs cumulatively contribute 90% of the standard deviation.” Then, Seurat v4.4.0 FindNeighbors and FindClusters functions were used to cluster similar ST spots [20]. Using significant PCs, Uniform Manifold Approximation and Projection (UMAP) was performed. The locations of the organizational tissue sections and UMAP clusters were visualized using the Seurat v4.4.0 SpatialPlot function. In addition, differential expression analysis was performed between clusters using the function Seurat v4.4.0 FindMarkers and Wilcoxon rank sum test. The top 100 genes were visualized by generating heat maps based on the largest absolute log twofold change (log2FC) values between the comparison groups.

### Cell-type enrichment analysis

Cell type enrichment was estimated across different spots in each of the Visium samples using Giotto v1.1.2 [21]. The public HGSOC scRNA-seq data (GSE180661) was used to identify the expression value of gene sets for the enrichment analysis [22]. A total of 322,649 cells (101,836 ovarian cancer cells, 55,656 fibroblasts, 5,550 endothelial cells, 67,607 T cells, 1,903 B cells, 6,150 plasma cells, 82,627 myeloid cells, 834 dendritic cells, and 486 mast cells) of fresh tissue samples acquired from 33 HGSOC patients with adnexa sites (ovary and fallopian tube) were included in the analysis. For each cell, the rank of the gene expression method was used, which requires an external scRNA-seq dataset as input, along with cell-type annotation. Ovarian cancer cells, fibroblasts, T cells, endothelial cells, myeloid cells, plasma cells, B cells, dendritic cells, and mast cells were annotated using the public HGSOC scRNA-seq data (GSE180661) [22]. Annotation information was used to calculate enrichment scores. For each cell type, genes were ranked based on fold-change values, while a relative gene ranking was determined for each spot [21]. Gene rankings were randomly shuffled 1,000 times to estimate the null distribution, and the null distribution was used to calculate the *p*-values for the enrichment scores [21]. Feature plots with enrichment scores were visualized using the R package Seurat v4.4.0 [18]. In addition, a heatmap was visualized through an enrichment score scaled by each cluster obtained by Visium expression data, and the cell type with the highest score was used to name the cluster. Therefore, the clusters of tumor tissues were classified into three regions: enriched regions of the tumor, stroma, and immune cells. Spearman’s correlation analysis between the cell type enrichment scores of cell populations was performed in the R and NR patients.

### Pathology annotations

Hematoxylin and eosin staining images of the HGSOC samples were read and annotated by an experienced pathologist. Each spot of the Visium data was annotated as a tumor or stroma spot using 10 × Genomics Loupe Browser 6.5.0. Immune spots were not annotated because of the presence of slide-frozen artifacts and the absence of tertiary immune structures. The annotated classification was compared with the computational clustering results of the Visium expression data.

### Gene set enrichment analyses (GSEA)

GSEA v4.3.2 (Broad Institute, Cambridge, MA, USA) was used for gene set enrichment analysis with HALLMARK gene sets from the molecular signature database (http://www.broadinstitute.org/gsea/msigdb) [23]. Pathways with normalized enrichment score (NES) > 0 were identified as upregulated and pathways with NES < 0 were identified as downregulated. Pathways with the top five NESs were selected to demonstrate each of the three regions.

### Copy number variation (CNV) analysis

CNV analysis was performed using SpatialInfrerCNV v0.1.0 [24]. Gene positions were extracted from the gtf file used for the mapping process. Gene expression estimation files from the Cell Ranger pipeline and histological annotation files of spatial barcodes with their corresponding regions (tumor, stroma, and immune) were imported. The annotation and gene expression estimation results were combined for each region, and the joined files with spots that passed the quality control filters were combined into a final matrix for downstream analysis. Copy number estimation was performed independently for each sample. For the SpatialInferCNV run function in the unsupervised analysis, the following parameters were used: cutoff = 0.1, cluster_by_groups = FALSE, denoise = TRUE, and HMM = FALSE. Then, the Seurat v4.4.0 SpatialPlot function was used to spatially visualize the unsupervised result [18]. In the supervised analysis, the remaining parameters other than cluster_by_groups = TRUE were the same as in the unsupervised analysis, which was visualized with a heat map classified by three regions.

### LR crosstalk analysis

To reveal LR interactions between regions where specific cell types were enriched, CellPhoneDB v5.0.0 was used for inference [25]. Only ligands and receptors expressed in more than 20% of cells in specific clusters were included in the analysis, and their mean values were calculated. Cellular crosstalk was determined based on significant enrichment of the reciprocal expression of ligands and receptors between clusters. Empirical shuffling was performed to estimate the ligands and receptors that represented significant cell-type specificity. To generate a null distribution of the mean expression of ligands and receptors, the random permutation of the cluster labels was performed. The percentage of mean values as high as or higher than the practical mean values was used to calculate the *p*-values for the specific cell type potential of the ligand and receptor pairs.

### CT acquisition and image analysis

CT phenotypes for radiogenomics were determined based on previous studies on HGSOC. The characteristics of the primary ovarian mass were recorded based on bilaterality, diameter (in case of bilateral ovarian masses, the larger ovarian mass was recorded), margin (smooth or irregular), composition (cystic/predominantly cystic, solid/predominantly solid), and calcification [9, 12, 17]. The larger diameter was assigned as positive if the diameter of the largest ovarian mass was equal to or greater than the cut-off value (median). In the presence of peritoneal carcinomatosis, the peritoneal seeding pattern was divided into predominant infiltrative or predominant nodular patterns. A predominantly infiltrative pattern was defined as the predominant appearance of ill-defined tumor involvement rather than definite nodular involvement. Seeding location (omentum, mesentery, pelvis, right upper quadrant, lesser sac, left upper quadrant, gastrohepatic, paracolic gutter, and gallbladder fossa) and the number of seeding locations were assessed. The presence, location, and number of metastatic LN were assessed. For further statistical analysis, continuous factors were categorized as binary factors based on the median or quartile cutoff values.

The CT phenotypes of both eight internal HGSOC samples and the 40 public HGSOC The Cancer Imaging Archive (TCIA) samples were categorized by a radiologist after reviewing the preoperative CT scans. Preoperative contrast-enhanced abdominal and pelvic CT scans were evaluated as follows. In eight internal HGSOC samples, CT image data were obtained using intra-institutional CT scanners (*n* = 4) (SOMATOM Definition AS + , Siemens Healthineers; Discovery CT750, GE Healthcare) and outside institutional CT scans (*n* = 4) (ECLOS, HITACHI or Lightspeed VCT, GE Healthcare or SOMATOM Definition AS, Seimens Healthineer). CT image data were acquired at 100–120 kVp and 97–294 reference mAs with a 5-mm thickness and no gap. For the intrainstitutional CT scan, 100–110 mL of the contrast material was injected, followed by a 20mL saline flush. In 40 TCIA cases, CT image data were obtained using CT scanners (LightSpeed Ultra, GE Healthcare; Lightspeed16, GE Healthcare; HiSpeed CT/i GE Healthcare; Sensation 16, Siemens; Sensation 64, Siemens) with 2–8 mm thickness.

### Radiogenomics analysis

The cell-type enrichment results were extracted for each tumor, stroma, and immune region, and DEG analysis was performed based on the two groups for each CT imaging factor classified by the radiologist. In total, 40 matched HGSOC patient with Public HGSOC bulk RNA-seq data from The Cancer Genome Atlas (TCGA) database and public CT image data from TCIA database were used to confirm our results. The differential expression of genes between the two groups of each CT phenotype was analyzed using edgeR v3.40.2 [26]. The log2FC of the gene expression between the two groups for each CT imaging factor was calculated. For the eight internal HGSOC samples, normalized ST data were analyzed using the FindMarkers function in Seurat v4.4.0 [18].

### Pathway enrichment analysis

Pathway enrichment analysis was performed using fgsea v1.24.0 by employing HALLMARK pathways [27]. The association score of the gene-CT phenotype for each gene was calculated, and the degree of overrepresentation was measured as NES. Pathways with a Benjamini–Hochberg adjusted *p*-value ≤ 0.05 were specified as significantly enriched pathways.

### Statistical analysis

Statistical analyses in the current study were performed using R software (R Foundation for Statistical Computing, Vienna, Austria, http://www.r-project.org). For differentially expressed gene analysis, *p*-values were determined by Wilcoxon rank sum test, and adjusted *p*-values were calculated based on Bonferroni correction using all features in the dataset. For enriched pathway analysis, *p*-values were determined by an adaptive multi-level split Monte-Carlo scheme, and adjusted *p*-values were calculated based on Benjamini–Hochberg procedure. An adjusted *p*-value of less than 0.05 was considered statistically significant.

## Results

### Patients’ characteristics

All eight patients with HGSOC had the stage-III-IV disease (Table 1). Among them, three were included in the NR group and five were included in the R group. The median follow-up period was 67 months (range, 40–90) for all patients; for the NR group, it was 78 months, whereas for the R group, this period was 60 months. The median initial CA125 level was 1,471 IU; for the NR group, it was 1,050 IU, whereas for the R group, this level was 1,893 IU. All patients received 6–9 cycles of platinum-based intravenous chemotherapy following debulking surgery. Of the four patients with BRCA1/2 mutations, three received maintenance therapy with a PARP inhibitor. One patient (NR3) did not receive this maintenance therapy, as there was no recurrence.

### Generation of the spatially resolved genomic profiles of HGSOC

Visium ST (10 × Genomics, Pleasanton, CA, USA) was used to investigate spatial gene expression. Spatial microarrays consisted of 4,992 unique barcoded spots, each with a diameter of 55 mm and a center-to-center distance of 100 mm, covering a capture area of 6.5 × 6.5 mm^2^. Each spot contained comprehensive transcriptome-wide information for all genes. The read count matrices were transformed into transcripts per kilobase of exome per million mapped reads.

**Fig. 1:**
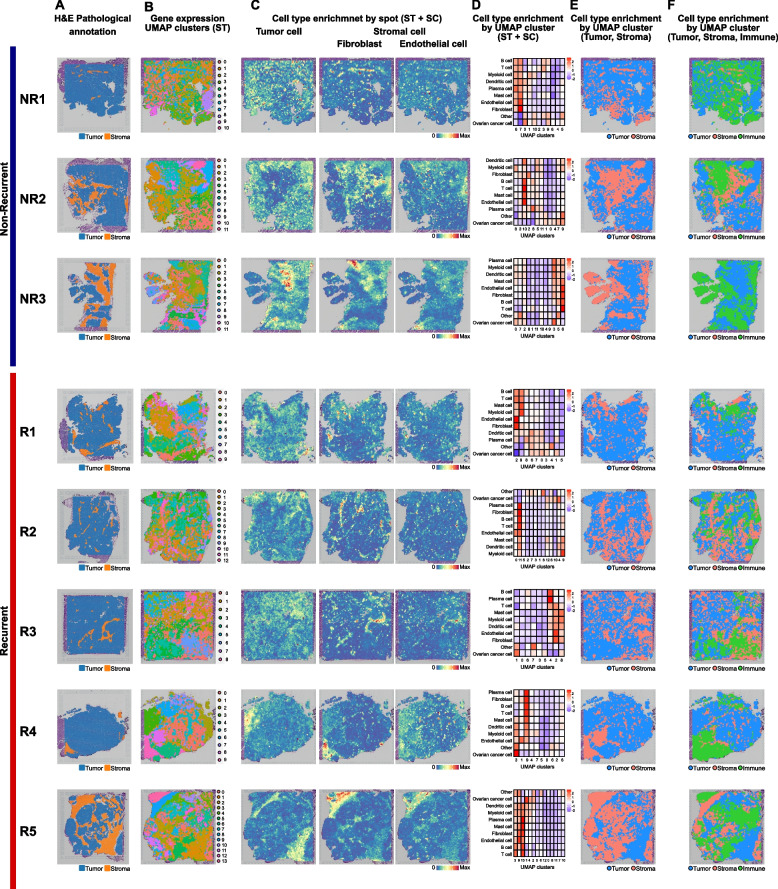
Characteristics of HGSOC using spatial transcriptomics. **A** Pathological annotation results of hematoxylin and eosin-stained tissue sections were conducted by a pathologist. **B** Gene expression-based clusters of tissue sections were identified by the UMAP-based dimensional reduction technique. **C** Cell type enrichment plot demonstrates the degree of enrichment of tumor and stromal cells. **D** Heatmap demonstrates cell type enrichment scores by the gene expression-based cluster. The x-axis represents clusters by gene expression pattern, and the y-axis represents the degree of cell type enrichment. **E** Organizational tissue sections separated by tumor and stromal regions. **F** Organizational tissue sections separated by tumor, stromal, and immune regions. ST = Spatial transcriptomics, SC = Single-cell RNA sequencing

**Fig. 2:**
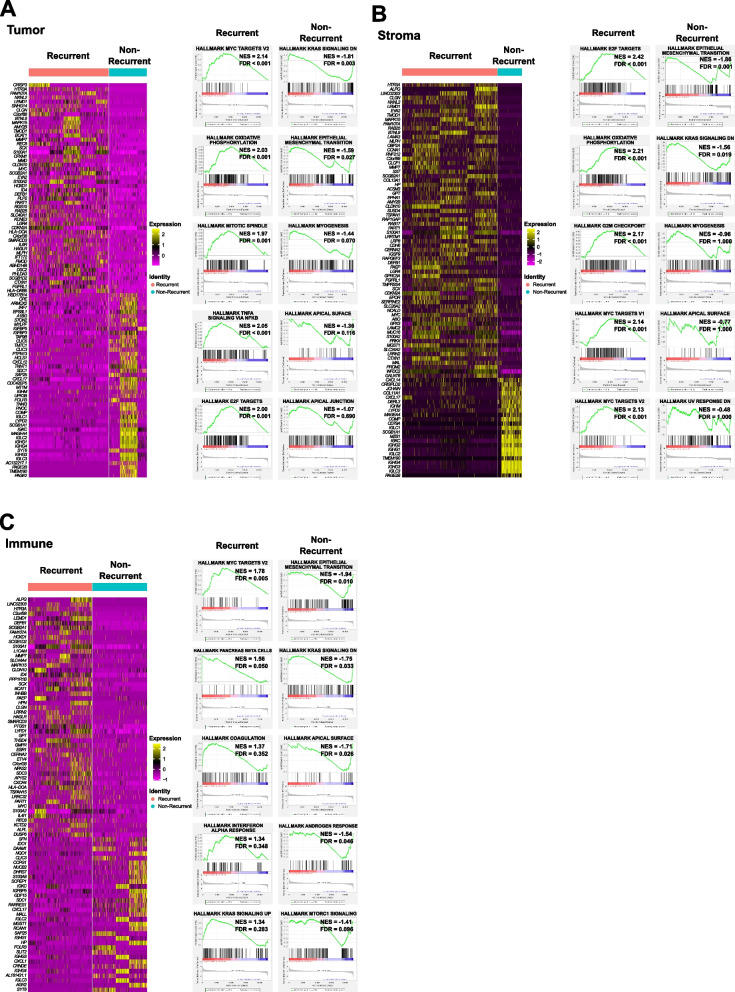
Heterogeneity associated with HGSOC. The heat map demonstrates the top 100 differentially expressed genes according to recurrence (adjusted *p*-value < 0.05 and log2FC > 2.0 or < -2.0). Columns represent the top 100 differentially expressed genes by tissue regions of (**A**) tumor, (**B**) stroma, and (**C**) immune. The enrichment plot indicates the top 5 hallmark pathways by tissue regions of (**A**) tumor, (**B**) stroma, and (**C**) immune

Differences in gene expression patterns between the R and NR groups were revealed by the Visium ST analysis (Figs. 1 and 2, Tables S1 and S2). Pathological annotation conducted by the pathologist was divided into tumor and stromal regions (Fig. 1A). Using the UMAP-based dimensional reduction technique, spatial clusters based on gene expression patterns were identified in each sample (Fig. 1B). The degree of cell type enrichment in tissue regions was identified using the expression profiles of HGSOC-specific cells using the public scRNA-seq data (GSE180661, Fig. 1C and D) [22]. Tumor region was defined as the area where tumor cell was enriched, the stroma region was defined as the area where stromal cells (fibroblasts and endothelial cells) were enriched, and the immune region was defined as the area where T, B, plasma, myeloid, dendritic, and mast cells were enriched (Fig. 1 and Fig. S1).

The clusters identified according to the spatial UMAP analysis were classified into two categories: tumor and stromal regions, and three categories: tumor, stromal, and immune regions, according to each tissue area, and visualized as UMAP (Fig. 1E and F). Figure 1E (two categories of tumor and stromal regions) was generated to compare the results of the pathological annotation. There was a partial concordance between the pathological annotation and spatial single-cell analysis (Fig. 1A, E, and F). In the NR1 patient, although there was a weak concordance with the spatial single-cell analysis result for the two categories of tumor and stromal regions (Fig. 1A and E), there was some degree of alignment with the three categories of tumor, stromal, and immune regions (Fig. 1A and F). Nine cell populations, including ovarian cancer cells, fibroblasts, endothelial cells, T cells, B cells, plasma cells, myeloid cells, dendritic cells, and mast cells, were identified. The identified cell clusters showed a high level of heterogeneity among the eight tumor samples. Interestingly, Spearman’s correlation analysis between cell type enrichment scores revealed co-localization in several cell populations (Fig. S2). In both the R and NR groups, negative correlations were observed between the ovarian cancer cell score and T and B cell scores, whereas positive correlations were evident in other cell types. The strongest correlation was observed between fibroblasts and endothelial cells, with correlations of 0.77 and 0.75 in the R and NR groups, respectively. Although the overall patterns were similar between the R and NR groups, positive correlations were identified in the R group between the ovarian cancer cell score and plasma cell score (correlation coefficient of 0.19) and between the ovarian cancer cell score and mast cell score (correlation coefficient of 0.12), whereas almost no correlations were found in the NR group. In summary, we identified morphologically distinct HGSOC tissue regions by performing ST analysis and indicated the heterogeneity of infiltrating cell populations.

### ST to reveal the TME of HGSOC

DEGs between HGSOC in the R group and the NR group were used to define statistically significant pathways with adjusted *p*-values < 0.05 and NES > 2.0, and the results were visualized according to tumor, stromal, and immune regions (Fig. 2). In the GSEA results, TNF-α signaling via NF-κB was significantly associated with genes upregulated only in the recurrent tumor region (Fig. 2A). In addition, oxidative phosphorylation and E2F targets were associated with upregulated genes only in the recurrent tumor and stromal regions, but not in the immune region (Fig. 2A and B). Moreover, the MYC target v1 was significant only in the recurrent stromal region (Fig. 2B). Our GSEA findings were consistent with previously identified pathways which are associated with the recurrence and poor prognosis of HGSOC [28–31]. In addition, *ERBB3* was downregulated in tumor (log2FC = 0.88, adjusted *p*-value ≤ 0.001), stromal (log2FC = 1.70, adjusted *p*-value ≤ 0.001), and immune (log2FC = 1.58, adjusted *p*-value ≤ 0.001) regions of the NR group (Table S2). *HMGA1* was also downregulated in stromal (log2FC = 0.66, adjusted *p*-value ≤ 0.001) and immune (log2FC = 0.33, adjusted *p*-value ≤ 0.001) regions of the NR group (Table S2). In summary, we observed that the GSEA and DEG results differed by the location of HGSOC tissues and demonstrated the potential role of spatial heterogeneity in promoting poor response to therapy and reducing the recurrence of HGSOC.

### CNV differences between spatial clusters

**Fig. 3:**
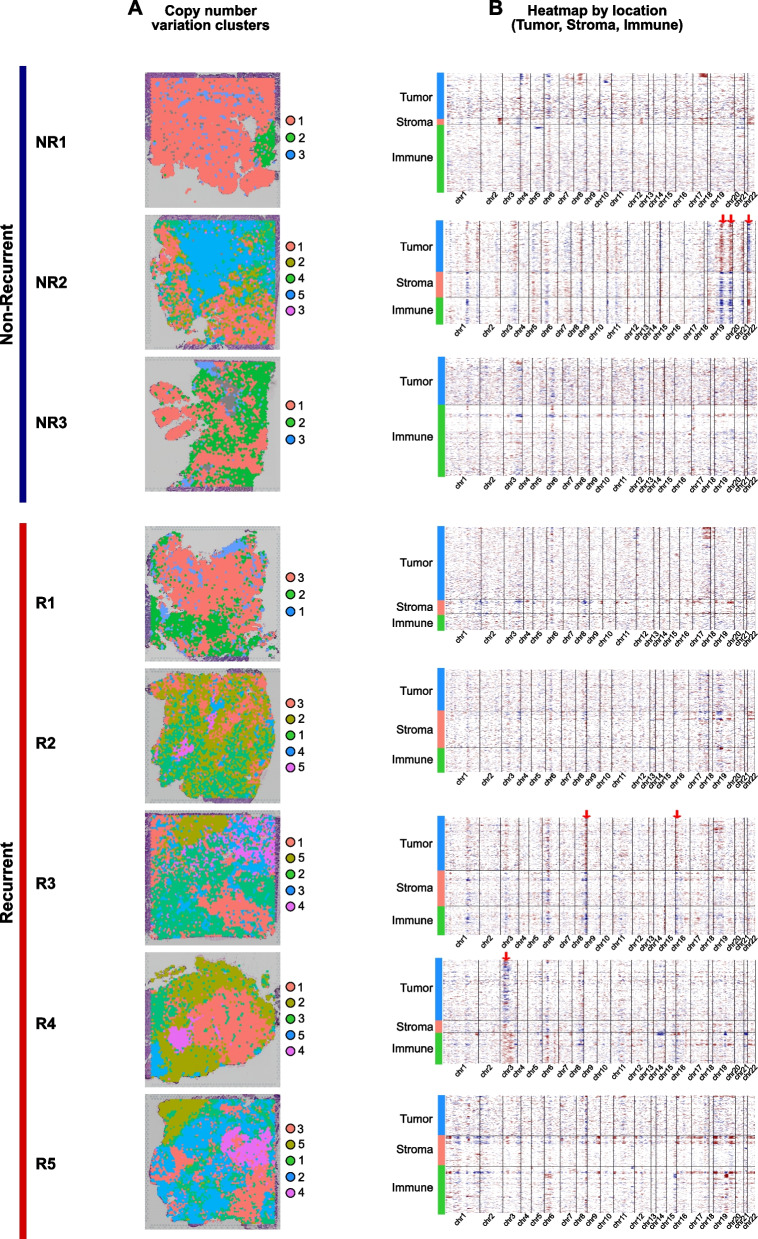
Distinct copy number variation patterns between spatial clusters. **A** Organizational tissue sections separated by copy number variation clusters according to the recurrent and non-recurrent patients. **B** The heat map indicates the rate of copy number variation in each recurrent and non-recurrent HGSOC sample. Columns represent the genomic region. The color key indicates the degree of score of copy number variation in direction to gain (red) or loss (blue). Each heat map was separated according to the tissue regions of the tumor, stroma, and immune using the color bars. Arrows indicate genomic regions representing different copy number variation profiles in the tumor, stromal, and immune regions

To reveal CNV differences between spatial clusters, CNV analysis was performed using expression data from the R and NR groups. The clustering of spots by CNV profiles was identified and visualized on the tissue images to compare the locations of the three HGSOC tissue regions (Figs. 1F and 3A). CNV clusters were consistent with clusters of the tumor, stroma, and immune regions. For example, in the R1 results, CNV cluster 1 (blue) was localized in the stromal region of the cell-type enrichment result. In addition, CNV cluster 4 (purple) was localized in the immune region, and CNV cluster 3 (orange) was localized in the cancer region in R5 patients. In the NR group, CNV cluster 3 (blue) was in agreement with the stromal region in NR1 patients, and CNV cluster 1 (orange) was in agreement with the immune region in NR3 patients. Interestingly, mixed regions were observed in the CNV clusters. In NR1 patients, CNV cluster 1 (orange) was localized in mixed regions (cancer and immune regions), whereas CNV cluster 2 (green) was localized in distinct tumor regions.

In addition, CNV profiles were compared between the three tissue regions, and distinct patterns were observed (Fig. 3B). In R3, amplification in the tumor region and deletion in the stroma and immune regions were observed on chromosomes 8 and 16, respectively. Deletion in the tumor region and amplification in the stromal and immune regions on chromosome 3 were observed in R4 patients. The findings from NR2 patients showed distinct amplification in the tumor region and deletion in the stromal and immune regions on chromosome 19, along with deletion in the tumor region and amplification in the stromal and immune regions on chromosome 22. These results provide evidence of different CNV profiles in the tumor, stromal, and immune regions. Interestingly, *HMGA1* was deleted in the tumor region of the NR group, and its protein level is known to be significantly increased in serous epithelial ovarian cancer (Table S3) [32]. Also, *ERBB3*, which was deleted in the immune region of the NR group, is known to be an oncogene, and its overexpression is correlated with the poor prognosis of patients with ovarian cancer (Table S3) [33–35]. In summary, we found that the CNV profiles of HGSOC showed heterogeneity not only in recurrent status but also in the location of the tissue.

### LR crosstalk heterogeneity to unravel characteristics of HGSOC

**Fig. 4:**
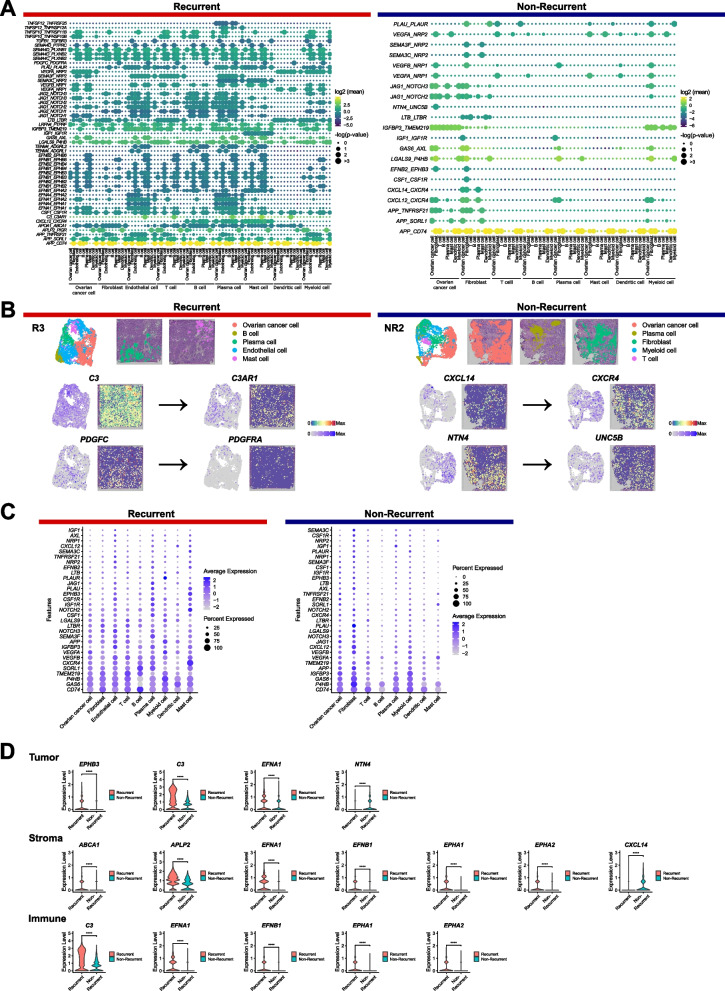
Differences of ligand-receptor crosstalk underlying HGSOC pattern. **A** The dot plot represents putative ligand–receptor interactions between spatial regions where each cell population is enriched. The size of the dot indicates the statistical significance of the indicated interactions. The color of the dot represents the means of the average expression level of the ligand and receptor from each interaction. **B** UMAP and spatial feature plots indicate the expression of genes specifically noted in the recurrent and non-recurrent patients, respectively (R3 and NR2). **C** The average expression of genes in the significant ligand–receptor pairs in both the recurrent and non-recurrent groups. **D** Violin plots represent the expression of genes, which are included in significant ligand–receptor crosstalk results and differentially expressed in recurrent or non-recurrent groups

LR heterogeneity between patients with R and NR HGSOC was revealed by crosstalk signaling network analysis to investigate whether specific signaling between specific cell populations affects the recurrence of HGSOC (Fig. 4 and Table S4). This analysis identified 51 distinct LR pairs in the R group and 21 pairs in the NR group. (Fig. 4A and Table S4). Of the 51 LR pairs, 32 (62.7%) were only noted in the R group and 19 (37.3%) were noted in both the R and NR groups. This overlap suggests that shared genes play crucial roles in the overall process of ovarian carcinogenesis. Unique LR pairs were identified in group R. Notably, pairs involved in signaling by TNF, specifically *TNFSF10*-*TNFRSF10B/TNFRSF11B* and *TNFSF12*-*TNFRSF25/TNFRSF12A*, were also observed in plasma cells (Fig. 4A and Table S4A). In addition, signaling by platelet-derived growth factor, galectin, complement, and amyloid-like protein was only significant in R patients, and signaling by netrin was only significant in NR patients (Fig. 4A and Table S4). Additionally, *C3*-*C3AR1* pairs involved in complement signaling were detected in plasma cells of the R group (Fig. 4A and B and Table S4A). Moreover, *PDGFC*-*PDGFRA* pairs, which are part of the platelet-derived growth factor signaling pathway, were identified in mast cells in the R group (Fig. 4A and B and Table S4A).

Within the NR group, a significant majority of interacting pairs (90.5%, *n* = 19/21) were also significant in the R group. The pairs *CXCL14*-*CXCR4* and *NTN4*-*UNC5B* were specifically noted in the NR group, and both are implicated in chemotaxis. *CXCL14* was exclusively identified in fibroblasts, whereas its corresponding receptor, *CXCR4*, was found in fibroblasts, ovarian cancer cells, and plasma cells (Fig. 4A and B, and Table S4B). *NTN4* was present in ovarian cancer cells, and its corresponding receptor, *UNC5B*, was identified in fibroblasts (Fig. 4A and B, and Table S4B). Notably, all chemotaxis-related crosstalk was observed in fibroblasts, highlighting the potential significance of the TME in these processes (Fig. 4A and Table S4B).

Based on the average expression of genes in the significant LR pairs in both the R and NR groups, eight genes, *CD74, P4HB, IGFBP3, GAS6, APP, TMEM219, VEGFA,* and *VEGFB*, were included in the top 10 genes common to both the R and NR groups (Fig. 4C). In addition, *ABCA1, EPHB3, APLP2, C3, EFNA1, EFNB1, EPHA1,* and *EPHA2* were differentially expressed in the R group compared with that in the NR group and were included in the significant LR pair only in the R group (Fig. 4D, Table S2, and Table S4). Interestingly, *ABCA1* (log2FC = 0.45, adjusted *p*-value < 0.001) and *APLP2* (log2FC = 0.62, adjusted *p*-value < 0.001) were significantly expressed in the stromal regions. *C3* was differentially expressed in the tumor (log2FC = 1.47, adjusted p-value < 0.001) and immune (log2FC = 1.09, adjusted *p*-value < 0.001) regions. *EPHA2* was differentially expressed in the stromal (log2FC = 0.81, adjusted *p*-value < 0.001) and immune regions (log2FC = 0.72, adjusted *p*-value < 0.001). In addition, *EFNA1* (log2FC > 0.52, adjusted *p*-value < 0.001) was differentially expressed in all three regions. *CXCL14* and *NTN4* were differentially expressed in the NR group compared with that in the R group and were included in the significant LR pair only in the NR group. *CXCL14* (log2FC =  − 2.73, adjusted *p*-value < 0.001) and *NTN4* (log2FC =  − 1.89, adjusted *p*-value < 0.001) were differentially expressed in stromal and tumor regions, respectively.

### DEGs according to recurrence status and CT phenotypes in spatial regions among eight internal HGSOC samples

We extracted six CT phenotypes from the eight internal HGSOC samples and the 40 public HGSOC TCIA and TCGA samples. Among them, two CT phenotypes were excluded because of the homogeneous CT phenotype of the R or NR groups in the eight internal HGSOC samples. Four CT phenotype were remained: larger diameter (≥ 7.8 cm), predominantly nodular seeding pattern, paracolic gutter seeding, and higher number of seeding locations (≥ 3). We hypothesized that opposite direction of regulated genes in the R and NR HGSOCs has tends to be associated with prognosis: genes upregulated in the R group compared with those in the NR group would be oncogenes, while genes upregulated in NR group compared with those in R group would be tumor suppresser genes. To verify the DEGs between the R and NR HGSOC according to the four CT phenotypes, only significant tumor-related genes revealed in previous studies with the largest absolute log2FC and opposite direction of regulation in the R and NR HGSOC were reported (Table 2).

**Table 2:** Differentially expressed genes relevant to recurrent status of eight internal HGSOC samples according to CT phenotypes in spatial regions

**CT phenotype**	**Gene name**	**Histologic subgroup**	**Recurrent group**			**Non-Recurrent group**		
			**Log2 FC**	***P*- value**	**Adjusted** ***p*-value**	**Log2 FC**	***P*- value**	**Adjusted** ***p*-value**
Characteristics of primary ovarian mass								
Larger diameter	*DAPL1*	Tumor	-0.91	< 0.001	< 0.001	1.29	< 0.001	< 0.001
	*RNASE1*	Stroma	-0.30	< 0.001	< 0.001	0.83	< 0.001	< 0.001
	*H19*	Tumor, Stroma, Immune	2.86, 2.89, 2.82	< 0.001, < 0.001, < 0.001	< 0.001, < 0.001, < 0.001	-3.35, -3.08, -2.89	< 0.001, < 0.001, < 0.001	< 0.001, < 0.001, < 0.001
	*S100A2*	Tumor, Stroma, Immune	2.49, 2.13, 2.50	< 0.001, < 0.001, < 0.001	< 0.001, < 0.001, < 0.001	-0.45, -0.79,-0.59	< 0.001, < 0.001, < 0.001	< 0.001, < 0.001, < 0.001
	*GPX3*	Tumor, Stroma, Immune	2.30, 2.31, 2.16	< 0.001, < 0.001, < 0.001	< 0.001, < 0.001, < 0.001	-0.90, -0.36, -1.12	< 0.001, < 0.001, < 0.001	< 0.001, 0.009, < 0.001
	*S100A6*	Tumor, Stroma, Immune	1.79, 1.91, 1.75	< 0.001, < 0.001, < 0.001	< 0.001, < 0.001, < 0.001	-0.54, -1.11, -1.01	< 0.001, < 0.001, < 0.001	< 0.001, < 0.001, < 0.001
Characteristics of peritoneal carcinomatosis								
Predominantly Nodular Seeding pattern	*CRIP1*	Tumor	1.83	< 0.001	< 0.001	-0.39	< 0.001	< 0.001
	*C20orf204*	Tumor, Stroma	1.42, 1.35	< 0.001, < 0.001	< 0.001, < 0.001	-0.92, -0.93	< 0.001, < 0.001	< 0.001, < 0.001
	*IGKC*	Tumor, Immune	1.42, 5.10	< 0.001, < 0.001	< 0.001, < 0.001	-4.27, -5.93	< 0.001, < 0.001	< 0.001, < 0.001
	*BCAT1*	Stroma	1.30	< 0.001	< 0.001	-0.29	< 0.001	0.001
	*PSMB9*	Stroma	-0.25	< 0.001	< 0.001	1.26	< 0.001	< 0.001
Paracolic gutter seeding	*C20orf204*	Tumor, Stroma	1.29, 1.32	< 0.001, < 0.001	< 0.001, < 0.001	-0.92, -0.93	< 0.001, < 0.001	< 0.001, < 0.001
	*IFI27*	Tumor, Stroma	0.26, 0.34	< 0.001, < 0.001	< 0.001, < 0.001	-1.91, -0.90	< 0.001, < 0.001	< 0.001, < 0.001
	*FXYD3*	Stroma	1.63	< 0.001	< 0.001	-0.39	< 0.001	0.001
	*BCAT1*	Stroma	1.24	< 0.001	< 0.001	-0.29	< 0.001	0.001
	*IGKC*	Immune	4.46	< 0.001	< 0.001	-5.93	< 0.001	< 0.001
	*IGHG1*	Immune	3.06	< 0.001	< 0.001	-5.20	< 0.001	< 0.001
	*HINT1*	Stroma	-0.60	< 0.001	< 0.001	1.07	< 0.001	< 0.001
Higher number of seeding locations ≥ 3	*C20orf204*	Tumor, Stroma	1.29, 1.32	< 0.001, < 0.001	< 0.001, < 0.001	-0.92, -0.93	< 0.001, < 0.001	< 0.001, < 0.001
	*IFI27*	Tumor, Stroma	0.26, 0.34	< 0.001, < 0.001	< 0.001, < 0.001	-1.91, -0.90	< 0.001, < 0.001	< 0.001, < 0.001
	*FXYD3*	Stroma	1.63	< 0.001	< 0.001	-0.39	< 0.001	0.001
	*BCAT1*	Stroma	1.24	< 0.001	< 0.001	-0.29	< 0.001	0.001
	*IGKC*	Immune	4.46	< 0.001	< 0.001	-5.93	< 0.001	< 0.001
	*IGHG1*	Immune	3.06	< 0.001	< 0.001	-5.20	< 0.001	< 0.001
	*HINT1*	Stroma	-0.60	< 0.001	< 0.001	1.07	< 0.001	< 0.001

*Abbreviation*: *Log2FC* Log twofold change

*P*-values were determined by Wilcoxon rank sum test

Adjusted *p*-values were calculated based on Bonferroni correction using all features in the dataset

In the R group, we detected 11 genes associated with tumor progression: *CRIP1, C20orf204, IGKC, H19, S100A2, GPX3, S100A6, IFI27, BCAT1, FXYD3,* and *IGHG1* (Table 2). *CRIP1* was specific to the tumor region, whereas *BCAT1* and *FXYD3* were confined to the stromal region. *IGKC* and *IGHG1* were expressed uniquely in the immune region. Furthermore, *C20orf204* and *IFI27* were expressed in both the tumor and stromal regions, highlighting their broad relevance across these areas. In the NR group, we identified four genes with roles in suppressing tumors in both the tumor and stromal regions (Table 2). *DAPL1* (larger diameter) was expressed in the tumor region, and *RNASE1* (larger diameter) was expressed in the stromal region [36–40]. Interestingly, these tumor-suppressing genes were related to immune modulation.

### DEGs according to CT phenotypes in spatial regions among eight internal HGSOC samples and 40 public HGSOC TCIA and TCGA samples

CT phenotypes of the eight internal HGSOC samples and the 40 public HGSOC TCIA and TCGA samples were evaluated (Table S5). To verify DEGs relevant to HGSOC according to CT phenotypes, genes with the standard of adjusted *p*-value less than 0.05 and the log2FC greater than 0.25 or less than − 0.25 overlapping both in the eight internal HGSOC samples and the 40 public HGSOC TCIA and TCGA samples have been revealed.

According to the CT phenotypes, 22 genes were differentially upregulated or downregulated in the tumor region, 24 in the stromal region, and 23 in the immune region. Of these, 15 genes have been reported to be associated with ovarian cancer in terms of differential expression, tumor growth, prognosis, and drug resistance [41–50]. Table 3 summarizes these DEGs according to CT phenotypes in a histologic subgroup.

**Table 3:** Differentially expressed genes relevant to eight internal HGSOC samples and 40 public HGSOC TCIA and TCGA samples according to the CT phenotypes in histologic subgroups

CT phenotype	Gene name	Histologic subgroup	Log2FC		*P*-value	Adjusted *p*-value
Characteristics of primary ovarian mass						
Bilaterality	*MGST1*	Stroma	0.54	< 0.001		< 0.001
Larger diameter	*SH3KBP1*	Stroma, immune	0.30, 0.37	< 0.001, < 0.001		< 0.001, < 0.001
	*FOXM1*	Stroma, immune	0.38, 0.50	< 0.001, < 0.001		< 0.001, < 0.001
	*SHKBP1*	Stroma, immune	0.30, 0.42	< 0.001, < 0.001		< 0.001, < 0.001
	*TCEAL2*	Stroma, immune	0.58, 0.54	< 0.001, < 0.001		< 0.001, < 0.001
	*MEG3*	Immune	0.42	< 0.001		< 0.001
Characteristics of peritoneal carcinomatosis						
Predominantly nodular seeding pattern	*CRISP3*	Tumor	-1.45	< 0.001		< 0.001
Paracolic gutter seeding	*DEFB1*	Tumor	0.28	< 0.001		< 0.001
	*ALPL*	Tumor	-0.48	< 0.001		< 0.001
Higher number of seeding locations ≥ 3	*PTGDS*	Tumor, stroma	-0.42, -0.31	< 0.001, < 0.001		< 0.001, < 0.001
	*ALPL*	Tumor	-0.48	< 0.001		< 0.001
Parietal peritoneal thickening	*LUM*	Tumor	0.37	< 0.001		< 0.001
	*MMP11*	Tumor, stroma, immune	0.70, 1.54, 0.67	< 0.001, < 0.001, < 0.001		< 0.001, < 0.001, < 0.001
	*DUSP1*	Tumor, immune	0.60, 0.90	< 0.001, < 0.001		< 0.001, < 0.001
	*CTSK*	Stroma	0.42	< 0.001		0.002
	*COL3A1*	Immune	0.42	< 0.001		< 0.001

*Abbreviation*: *Log2FC* Log twofold change

*P*-values were determined by Wilcoxon rank sum test

Adjusted *p*-values were calculated based on Bonferroni correction using all features in the dataset

For each of 40 matched HGSOC patients, CT phenotypes were extracted from TCIA database and bulk RNA sequencing data were extracted from TCGA database

**Fig. 5:**
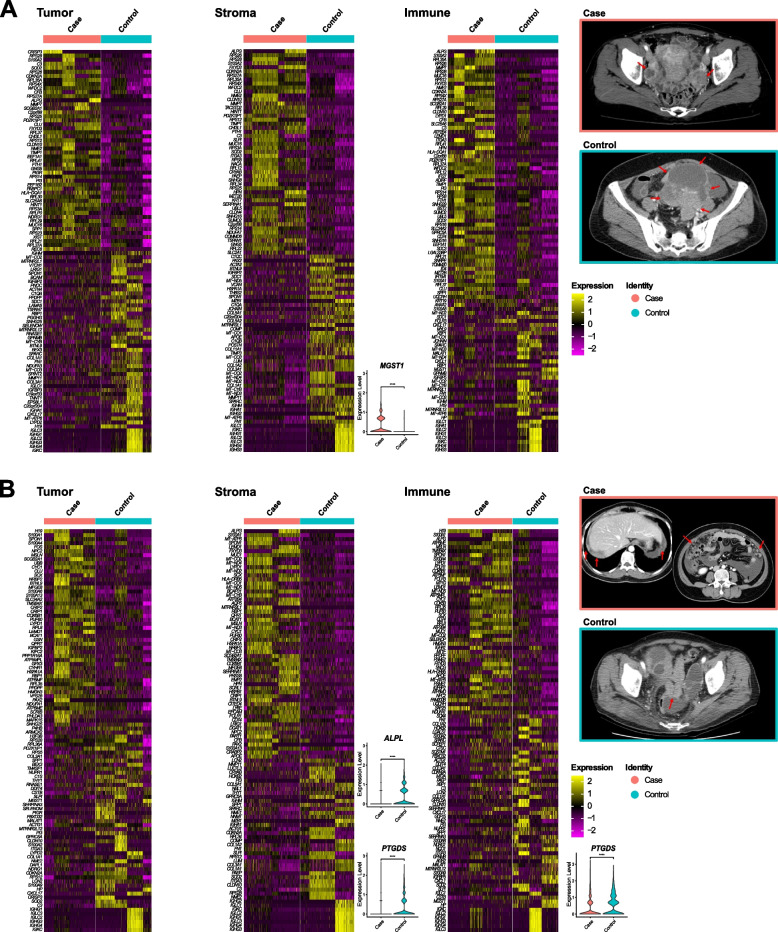
Radiogenomics profiling between CT phenotypes and spatial transcriptomic patterns. Patterns of the case and control CT phenotypes are shown: (**A**) bilaterality of ovarian mass (case; red bar) and unilaterality of ovarian mass (control; blue bar), (**B**) higher number of location (≥ 3) (case; red bar) and lower number of location (< 3) (control; blue bar). The heat map demonstrates the top 100 differentially expressed genes according to each CT phenotype from eight internal HGSOC samples (adjusted *p*-value < 0.05 and log2FC > 2.0 or <  − 2.0). Columns represent the top 100 differentially expressed genes. The color key indicates the degree of differential gene expression in either direction to upregulation (yellow) or downregulation (purple). Violin plots represent differentially expressed genes relevant to the CT phenotypes of both eight internal HGSOC and 40 public HGSOC TCIA and TCGA samples. The CT examples with a red border indicates case and the CT example with a blue border indicates control of each CT phenotypes. **A** Bilateral ovarian mass (arrows) in case example. Unilateral ovarian mass (arrows) in control example. **B** Three locations of seeding metastases (arrows) at right upper quadrant, left upper quadrant, and omentum in case example. Single location of seeding metastasis at pelvic cavity (arrows) in control example

Six CT phenotypes had differentially expressed genes: bilaterality of ovarian mass, larger diameter (≥ 7.8 cm), predominantly nodular seeding pattern, paracolic gutter seeding, a higher number of seeding locations (≥ 3), and peritoneal thickening. The spatial transcriptomic pattern of the eight internal HGSOC samples showed DEGs according to different CT phenotypes especially in bilaterality and higher number of seeding locations (Fig. 5).

The predominantly nodular seeding pattern showed the downregulation of *CRISP3* in the tumor region (log2FC =  − 1.45, adjusted *p*-value < 0.001, Table 3). The higher number of seeding locations (≥ 3) showed the downregulation of *PTGDS* in the tumor (log2FC =  − 0.42, adjusted *p*-value < 0.001) and stromal regions (log2FC =  − 0.31, adjusted *p*-value < 0.001) and *ALPL* in the tumor region (log2FC =  − 0.48, adjusted *p*-value < 0.001, Table 3). *ALPL* was also downregulated in the CT phenotypes of paracolic gutter seeding (log2FC =  − 0.48, adjusted *p*-value < 0.001, Table 3). Parietal peritoneal thickening showed the overexpression of *LUM*, *MMP11*, and *DUSP1* in the tumor region, *MMP11* and *CTSK* in the stromal region, and *MMP11* and *CTSK* in the immune region (adjusted *p*-values ≤ 0.002, Table 3).

### Enriched pathway analysis of the DEGs according to CT phenotypes among eight internal HGSOC samples

The CT phenotypes of paracolic gutter seeding, higher number of seeding locations, and parietal peritoneal thickening were significantly associated with enriched pathways in the eight internal HGSOC samples (Table 4). These were also the significant DEG-related CT phenotypes in the eight internal HGSOC samples and the 40 public HGSOC TCIA and TCGA samples (Table 3).

**Table 4:** Enriched pathway analysis of the differentially expressed gene according to the CT phenotypes among eight internal HGSOC samples

**CT phenotype**	**Histologic subgroup**	**Pathway**	*P*-value	**Adjusted** *p*-value
Paracolic gutter seeding	Stroma	HALLMARK_OXIDATIVE_PHOSPHORYLATION	0.002	0.050
		HALLMARK_EPITHELIAL_MESENCHYMAL_TRANSITION	0.002	0.050
	Immune	HALLMARK_TNFA_SIGNALING_VIA_NFKB	0.002	0.045
		HALLMARK_EPITHELIAL_MESENCHYMAL_TRANSITION	0.002	0.045
Higher number of seeding locations ≥ 3	Stroma	HALLMARK_OXIDATIVE_PHOSPHORYLATION	0.002	0.050
		HALLMARK_EPITHELIAL_MESENCHYMAL_TRANSITION	0.002	0.050
	Immune	HALLMARK_TNFA_SIGNALING_VIA_NFKB	0.002	0.045
		HALLMARK_EPITHELIAL_MESENCHYMAL_TRANSITION	0.002	0.045
Parietal peritoneal thickening	Stroma	HALLMARK_MYC_TARGETS_V1	0.004	0.026
		HALLMARK_EPITHELIAL_MESENCHYMAL_TRANSITION	0.006	0.031
		HALLMARK_GLYCOLYSIS	0.004	0.026
		HALLMARK_IL6_JAK_STAT3_SIGNALING	0.006	0.031
		HALLMARK_ALLOGRAFT_REJECTION	0.002	0.018
		HALLMARK_E2F_TARGETS	0.002	0.018
		HALLMARK_COMPLEMENT	0.002	0.018
		HALLMARK_G2M_CHECKPOINT	0.002	0.018
		HALLMARK_INTERFERON_GAMMA_RESPONSE	0.002	0.018
		HALLMARK_TNFA_SIGNALING_VIA_NFKB	0.002	0.018
	Immune	HALLMARK_INFLAMMATORY_RESPONSE	0.002	0.026
		HALLMARK_ALLOGRAFT_REJECTION	0.002	0.026
		HALLMARK_G2M_CHECKPOINT	0.004	0.037
		HALLMARK_TNFA_SIGNALING_VIA_NFKB	0.002	0.026
		HALLMARK_MYC_TARGETS_V1	0.003	0.026
		HALLMARK_GLYCOLYSIS	0.002	0.026

*P*-values were determined by an adaptive multi-level split Monte-Carlo scheme

Adjusted *p*-values were calculated based on Benjamini–Hochberg procedure

Oxidative phosphorylation was significantly enriched according to the CT phenotype of paracolic gutter seeding and the higher number of seeding locations in the stromal region (Table 4). TNF-α signaling via NF-κ was significantly enriched according to the paracolic gutter seeding and higher number of seeding locations in the stromal region and parietal peritoneal thickening in the stromal and immune regions (Table 4). The G2/M checkpoint, E2F target, and MYC target V1 were significantly enriched in the parietal peritoneal thickening of the stromal and immune regions (Table 4). These pathways were also the top five enriched pathways in the R group of the eight internal HGSOC samples (Fig. 2): oxidative phosphorylation in the tumor and stromal regions, TNF-α signaling via NF-κ in the tumor region, G2/M checkpoint, E2F target, and MYC target V1 in the stromal region.

## Discussion

Unveiling the distinct components of a highly complex TME is crucial for developing optimal therapeutic strategies for recurrent HGSOC [51, 52]. However, the underlying spatial landscape of recurrent HGSOC is poorly understood. In this study, we identified novel molecular markers indicative of the spatial heterogeneity of HGSOC, which could potentially serve as markers of favorable or poor prognosis, using radiogenomics with ST and CT imaging for the first time. First, the ST study revealed heterogeneity between the R and NR groups in different spatial regions. Second, we identified distinct markers associated with several CT phenotypes using the eight internal HGSOC samples and the 40 public HGSOC TCIA and TCGA samples. Lastly, we revealed that several CT phenotypes were associated with poor prognosis by identifying pathways that were commonly enriched in the CT phenotype and R group. This comprehensive study revealed a new perspective on key markers for revealing the distinct signatures of HGSOC according to tissue region, recurrence status, CNV, LR crosstalk, and CT phenotype.

In the current ST study, we identified prognostic markers that exhibited common or distinct significance between the R and NR groups. Among the genes that were highly expressed and commonly significant LR pairs in both the R and NR groups, *CD74*, *GAS6*, and *VEGFA* were associated with carcinogenesis and were prominent markers of targeted therapy in ovarian cancer [53–58]. To further investigate the heterogeneity of recurrence status in different spatial regions, DEGs in the R group compared with those in the NR group and those included in the significant LR pair only in the R group were identified according to spatial regions. Interestingly, previous studies revealed that the high expression of *ABCA1*, *APLP2*, *C3*, *EPHA2*, and *EFNA1* were associated with poor prognosis and epithelial ovarian cancer progression [59–64]. Unique LR pairs identified in group R were also known to be associated with recurrence and progression of ovarian cancer. According to a recent study, patients with recurrent ovarian cancer exhibit increased levels of interleukin-6 and TNF-α in serum compared with levels in patients with NR ovarian cancer [65]. TWEAK, a member of the TNF ligand family, is also reported to induce angiogenesis in vivo [66]. These results explain why signaling by TNF is associated with the recurrence of HGSOC [67]. The complement system, a key regulator of the TME in cancer immunity, promotes chronic inflammation, an immunosuppressive microenvironment, angiogenesis, and cancer-related signaling. Complement effectors, C1q or anaphylatoxins C3a and C5a, and their receptors, C3aR and C5aR1, play a prominent role in ovarian cancer progression [68]. In addition to two genes (*CXCL14* and *NTN4*) that were differentially expressed in the NR group compared with that in the R group, those included in the significant LR pair only in the NR group were associated with a good prognosis of ovarian or other gynecological cancers [67, 69–72]. Interestingly, the high expression of *CXCL14*, a powerful angiostatic chemokine that inhibits angiogenesis, is correlated with good progression-free survival in endometrioid ovarian cancer and is expressed in normal tissues such as epithelia and ovarian cancer stroma [67, 69, 70, 73]. Consistent with previous studies, we observed that *CXCL14* was differentially expressed in the NR group in the stromal region and highly expressed in stromal cells. Moreover, we observed the downregulation and copy number deletion of two genes (*ERBB3* and *HMGA1*) in the NR group, which are associated with poor prognosis and tumorigenesis in ovarian cancer. Taken together, our findings suggest potential tumor-suppressive and tumor-promoting markers in HGSOC and further corroborate that the significance of previously identified prognostic markers differed according to the location of the ovarian cancer tissue.

With respect to radiogenomics, similar to the present study, many studies revealed that the margin and composition of primary ovarian mass were not associated with the genetic types of HGSOC [9, 12, 17]. The extent and location of peritoneal seeding rather than the characteristics of primary ovarian mass are associated with the gene type of HGSOC [9, 12]. Our study revealed that the higher number of seeding locations (≥ 3) was associated with *PTGDS* downregulation in both the tumor and stromal regions in the eight internal HGSOC samples and the 40 public HGSOC TCIA and TCGA samples. Additionally, *PTGDS* was one of the DEGs that was upregulated only in the NR group in the tumor and stromal regions of the eight internal HGSOC samples. PGD2, the protein product of *PTGDS*, inhibits in vitro and in vivo ovarian cancer cell growth and prolongs the survival time of nude mice in serous ovarian cancer [74]. Moreover, Alves et al. revealed that PGD2 was associated with a good prognosis in patients with HGSOC [45]. Lesser peritoneal seeding burden and extent are good prognostic factors in HGSOC [75, 76]. Thus, *PTGDS* is associated with less peritoneal tumor spread and a good prognosis in HGSOC.

TNF-α signaling via NF-κB, oxidative phosphorylation, G2/M checkpoint, E2F target, and MYC target v1 signaling were common pathways that were significantly enriched in both the R group and certain CT phenotypes. TNF-α signaling is associated with a high rate of metastasis and recurrence owing to the residual population of cancer stem cells (CSCs) [28]. Oxidative phosphorylation is a key metabolic process within cells, which plays a pivotal role in promoting resistance to cancer drugs and markedly affects responses to anticancer therapy [29]. After exposure to cytotoxic agents, a minimal residual cancer cell population may enrich CSCs contributing to cancer recurrence as CSCs exhibit an increased oxidative phosphorylation metabolic signature, thereby rendering them insensitive to cancer treatment [29]. In recent studies, the E2F signaling pathway and high E2F scores, which are based on the expression of the E2F target geneset, were associated with metastasis, poor prognosis, and recurrence of cancers such as breast and prostate cancer [77, 78]. Moreover, a previous study revealed that the MYC target v1, which is associated with cell proliferation, was positively correlated with ovarian cancer [79]. More than half of patients with HGSOC exhibit MYC oncogenic pathway activation, and MYC amplification is associated with tumor recurrence [80–82].

Our study revealed that several CT phenotypes were related to the upregulation of genes with poor prognosis and the downregulation of genes with favorable prognosis: bilaterality, predominantly nodular seeding pattern, paracolic gutter seeding, higher number of seeding locations (≥ 3), and parietal peritoneal thickening. Additionally, some of these CT phenotypes shared enriched pathways related to poor prognosis in the R group and contained DEGs highly expressed in the R group or low expressed in the NR group. Interestingly, paracolic gutter seeding and a higher number of seeding locations were common CT phenotypes in all results. According to the revised International Federation of Gynecology and Obstetrics (FIGO) staging system, the bilaterality of ovarian cancer, tumor involvement within or outside the pelvis, microscopic or macroscopic extrapelvic peritoneal involvement, and peritoneal tumors larger than 2 cm are the major factors that determine the stage and affect the patient’s prognosis. A predominantly nodular seeding pattern and parietal peritoneal thickening were associated with macroscopic peritoneal involvement and peritoneal tumor size. Paracolic gutter seeding and a higher number of locations were also associated with extrapelvic extension. Thus, the CT phenotypes revealed in the present study were associated with poor prognosis and could be explained by FIGO staging. Using ST, this study identified meaningful CT phenotypes and genes associated with prognosis that could be key to targeted therapies. Further research is required to verify these findings.

To the best of our knowledge, the present study is the first to demonstrate that comprehensive radiogenomics using ST and CT imaging can improve the prognosis of HGSOC. Nevertheless, the present study has several limitations. First, the small sample size of patients with HGSOC (three NR samples and five R samples) may have led to our findings being underpowered. Owing to a 5-year survival rate of less than 35% and a recurrence rate of about 80% after the diagnosis of patients with HGSOC, it was challenging to collect samples from patients with stage III-IV non-recurrent HGSOC [3, 83, 84]. To increase the reliability of our results, we verified our findings using the public HGSOC bulk RNA-seq data from TCGA and the public HGSOC CT image data from TCIA (*n* = 40). In addition, we plan to expand our cohort in future studies. Secondly, the limited resolution allows for analysis with a restricted number of cells in each spot. However, an integration analysis with the public HGSOC scRNA-seq data (322,649 cells) was performed to determine the degree of enrichment of HGSOC-specific cell populations at the single-cell level [22]. Upcoming advanced whole-transcriptome spatial technology for single-cell-scale resolution based on the microscopic view of tissues may uncover intratumoral heterogeneity with greater efficacy [85]. Third, the CT phenotype results were only recorded by one radiologist, which is a limitation that ignores the possibility of inter-reader disagreement. However, given the simple nature of this imaging analysis, one radiologist was sufficient.

## Conclusion

Taken together, our findings provide new evidence on a spatial landscape and heterogeneous TME that may affect HGSOC recurrence or non-recurrence within 5 years. Our results will inspire researchers to incorporate spatial landscapes and CT phenotypes into the investigations of various factors affecting the prognosis and treatment of HGSOC.

### Supplementary Information

Supplementary Material 1: Fig. S1. Cell type enrichment results of nine cell populations. Cell type enrichment plots demonstrate the degree of enrichment of nine cell populations in each patient.

Supplementary Material 2: Fig. S2. Spearman correlation results between cell type enrichment scores. The Spearman correlation heatmap demonstrates significant statistical correlation values between cell type enrichment scores. Blue squares indicate positive correlations and red squares indicate negative correlations

Supplementary Material 3: Table S1. Differentially expressed genes in each cluster

Supplementary Material 4: Table S2. Differentially expressed genes in recurrence of HGSOC

Supplementary Material 5: Table S3. Copy number variation from spatially resolved mRNA profiles in HGSOC

Supplementary Material 6: Table S4A. Significant ligand-receptor crosstalk of recurrent HGSOC. Table S4B. Significant ligand-receptor crosstalk of non-recurrent HGSOC

Supplementary Material 7: Table S5. CT phenotypes of eight internal HGSOC samples and 40 public HGSOC TCIA and TCGA samples

## Data Availability

The ST data generated in this study is available in the Gene Expression Omnibus (GEO) database under accession code GSE263920. The internal CT imaging used in this study is not publicly available due to the restrictions of hospital regulations and patient privacy. Restricted access for this data supporting the findings of our study can be obtained via requests for non-commercial purposes from the corresponding authors Dr. Mi-Ryung Han (genetic0309@inu.ac.kr) or Dr. Youn Jin Choi (yunno@catholic.ac.kr) typically within one month. The public HGSOC scRNA-seq dataset reused in this study is available in the GEO database under accession code GSE180661. The public HGSOC bulk RNA-seq data and the public HGSOC CT image data are available in the TCGA (https://portal.gdc.cancer.gov/) and TCIA (https://www.cancerimagingarchive.net/) database, respectively. The code for the bioinformatic analysis has been uploaded to GitHub (https://github.com/jhyhy0825/ST_HGSOC).

## Abbreviations

AJCC: American Joint Committee on Cancer
CNV: Copy number variation
CSCs: Cancer stem cells
CT: Computed tomography
DEGs: Differentially expressed genes
FIGO: International Federation of Gynecology and Obstetrics
HGSOC: High-grade serous ovarian cancer
log2FC: Log twofold change
LR: Ligand-receptor
NES: Normalized enrichment score
NR: Non-recurrent
PC: Principal component
R: Recurrent
scRNA-seq: Single-cell RNA sequencing
ST: Spatial transcriptomics
TCGA: The Cancer Genome Atlas
TCIA: The Cancer Imaging Archive
TME: Tumor microenvironment
TNF: Tumor necrosis factor
UMAP: Uniform Manifold Approximation and Projection
