# Supplementary material for: Integrated analysis of spatial transcriptomics and CT phenotypes for unveiling the novel molecular characteristics of recurrent and non-recurrent high-grade serous ovarian cancer
Source: Biomark Res. 2024 Aug 12;12:80. doi: 10.1186/s40364-024-00632-7 (PMC11318304; doi:10.1186/s40364-024-00632-7)
Supplement: Supplementary file 1 — Supplementary Material 1: Fig. S1. Cell type enrichment results of nine cell populations. Cell type enrichment plots demonstrate the degree of enrichment of nine cell populations in each patient. [file 40364_2024_632_MOESM1_ESM.pdf]

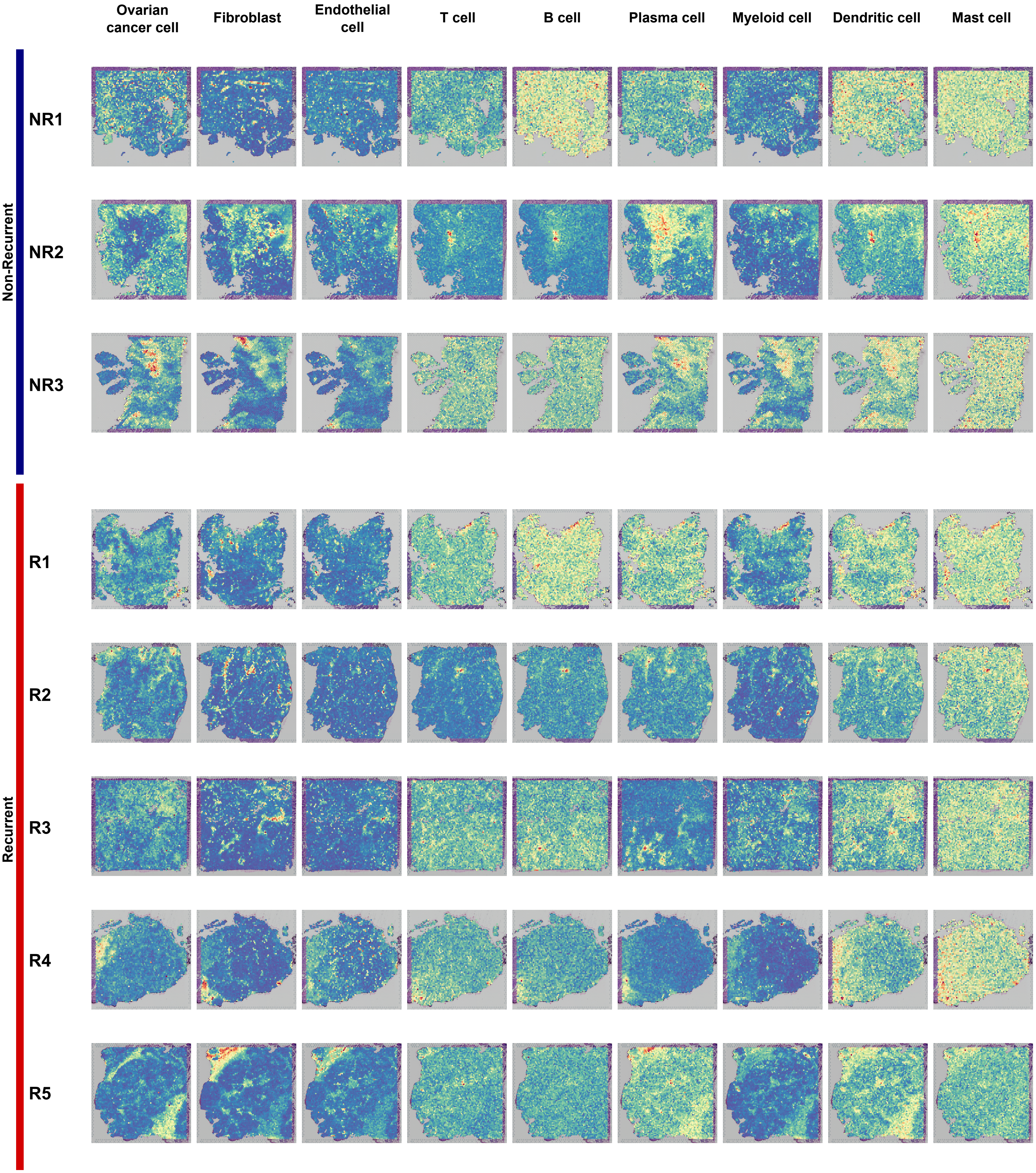

Fig. S1 | Cell type enrichment results of nine cell populations. Cell type enrichment plots demonstrate the degree of enrichment of nine cell populations in each patient.
