# Supplementary material for: Integrated analysis of spatial transcriptomics and CT phenotypes for unveiling the novel molecular characteristics of recurrent and non-recurrent high-grade serous ovarian cancer
Source: Biomark Res. 2024 Aug 12;12:80. doi: 10.1186/s40364-024-00632-7 (PMC11318304; doi:10.1186/s40364-024-00632-7)
Supplement: Supplementary file 2 — Supplementary Material 2: Fig. S2. Spearman correlation results between cell type enrichment scores. The Spearman correlation heatmap demonstrates significant statistical correlation values between cell type enrichment scores. Blue squares indicate positive correlations and red squares indicate negative correlations [file 40364_2024_632_MOESM2_ESM.pdf]

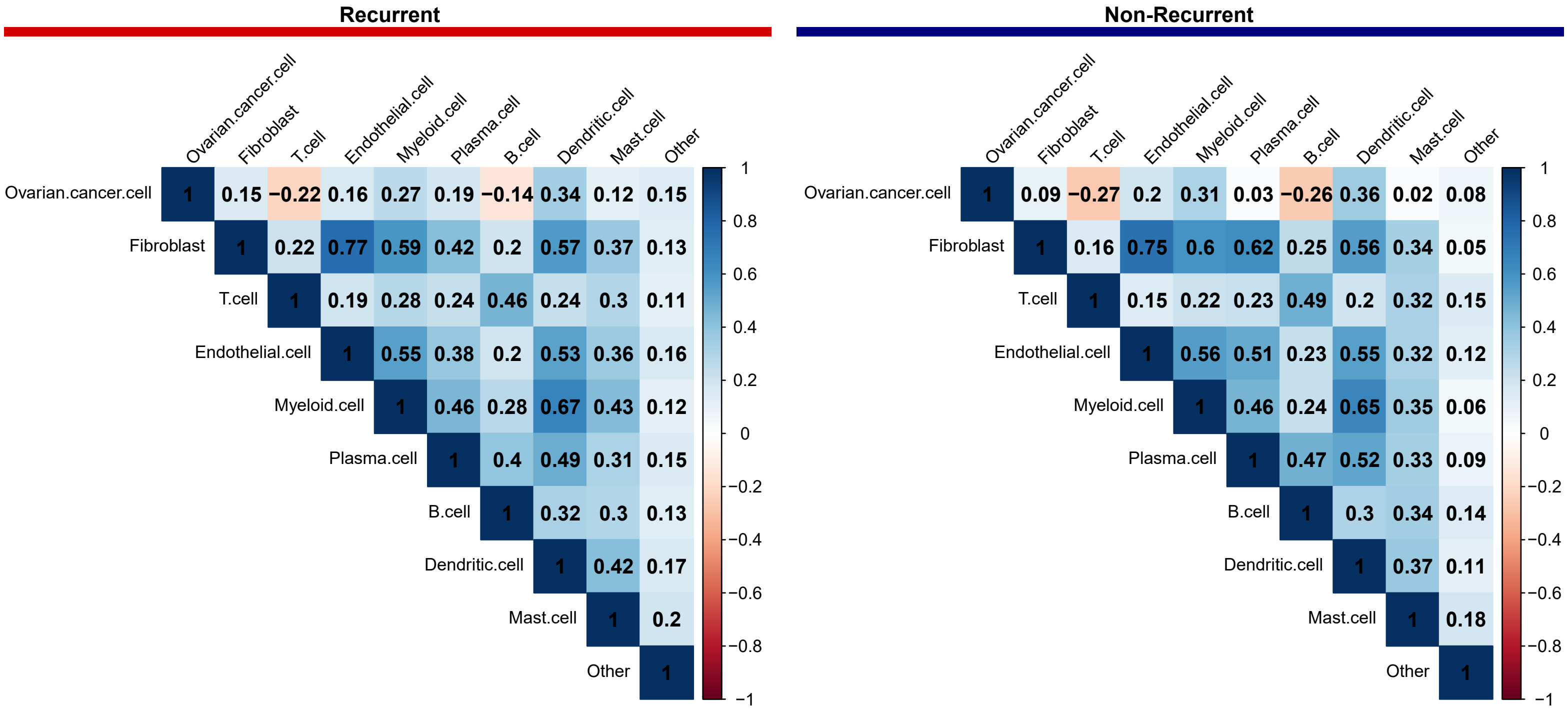

Fig. S2 | Spearman correlation results between cell type enrichment scores. The Spearman correlation heatmap demonstrates significant statistical correlation values between cell type enrichmnet scores. Blue squares indicate positive correlations and red squares indicate negative correlations.
